# A stapled peptide mimetic of the CtIP tetramerization motif interferes with double-strand break repair and replication fork protection

**DOI:** 10.1126/sciadv.abc6381

**Published:** 2021-02-19

**Authors:** Anika Kuster, Nour L. Mozaffari, Oliver J. Wilkinson, Jessica L. Wojtaszek, Christina Zurfluh, Sara Przetocka, Dawid Zyla, Christine von Aesch, Mark S. Dillingham, R. Scott Williams, Alessandro A. Sartori

**Affiliations:** 1 Institute of Molecular Cancer Research, University of Zürich, Winterthurerstrasse 190, CH-8057 Zürich, Switzerland.; 2 School of Biochemistry, University of Bristol, University Walk, Clifton BS8 1TD, Bristol, UK.; 3 Structural Cell Biology Group, Genome Integrity and Structural Biology Laboratory, National Institute of Environmental Health Sciences, U.S. National Institutes of Health, Department of Health and Human Services, Research Triangle Park, NC 27709, USA.; 4 Institute of Molecular Biology and Biophysics, ETH-Zürich, Otto-Stern-Weg 5, CH-8093 Zürich, Switzerland.

## Abstract

Cancer cells display high levels of DNA damage and replication stress, vulnerabilities that could be exploited by drugs targeting DNA repair proteins. Human CtIP promotes homology-mediated repair of DNA double-strand breaks (DSBs) and protects stalled replication forks from nucleolytic degradation, thus representing an attractive candidate for targeted cancer therapy. Here, we establish a peptide mimetic of the CtIP tetramerization motif that inhibits CtIP activity. The hydrocarbon-stapled peptide encompassing amino acid residues 18 to 28 of CtIP (SP^18–28^) stably binds to CtIP tetramers in vitro and facilitates their aggregation into higher-order structures. Efficient intracellular uptake of SP^18–28^ abrogates CtIP localization to damaged chromatin, impairs DSB repair, and triggers extensive fork degradation. Moreover, prolonged SP^18–28^ treatment causes hypersensitivity to DNA-damaging agents and selectively reduces the viability of *BRCA1*-mutated cancer cell lines. Together, our data provide a basis for the future development of CtIP-targeting compounds with the potential to treat patients with cancer.

## Teaser

A stapled peptide targets CtIP tetramers to inhibit DNA repair and exert synthetic lethality in BRCA1-mutant cancer cells.

## INTRODUCTION

DNA double-strand breaks (DSBs) are the most cytotoxic lesions induced by ionizing radiation and certain chemotherapeutic agents. In contrast to normally dividing cells, hyperproliferating cancer cells are commonly exposed to higher loads of DSBs due to oncogene-induced replication stress and inherited or acquired defects in components of the DNA damage response machinery (*1*). Consequently, efficient DSB repair is pivotal for cancer progression and frequently associated with therapy resistance (*2*). DSBs are mainly repaired by classical nonhomologous end joining (c-NHEJ) and homologous recombination (HR) in an error-free manner. Otherwise, DSBs with flanking homologous sequences can be subjected to mutagenic pathways, including alternative end joining (a-EJ) (*3*).

The human CtIP protein, in conjunction with the MRE11-RAD50-NBS1 (MRN) nuclease complex, plays a critical role in DSB repair pathway choice through promoting DNA end resection, the initial step in HR (*4*). A-EJ, which is frequently found up-regulated in HR-deficient tumors associated with *BRCA1* mutations, equally relies on CtIP-dependent end resection to expose microhomology regions used for annealing and repair of DSBs (*5*). Intriguingly, a study using mouse models of human breast cancer proposed that CtIP could promote tumorigenesis by facilitating a-EJ–dependent chromosomal instability (*6*). We have recently shown that CtIP protects nascent DNA at stalled replication forks from nucleolytic degradation by DNA2 (*7*). Moreover, we revealed that CtIP acts independently from BRCA1 in fork protection and that loss of CtIP in BRCA1-deficient cells aggravates replication stress–induced genomic instability, ultimately leading to increased cell death. On the basis of these findings, targeted inhibition of CtIP in *BRCA1*-mutated breast and ovarian cancers might represent a promising therapeutic approach (*7*).

Over the past decade, CtIP has emerged as a polyvalent adaptor protein containing several short linear sequence motifs implicated in protein-protein interactions. The overall sequence similarity between CtIP counterparts of different species is extremely poor, with the most conserved regions located at the N and C terminus. The N-terminal domain (NTD) harbors a coiled-coil region implicated in CtIP oligomerization (*8*–*10*); meanwhile, the C-terminal domain (CTD) facilitates DNA binding and MRN interaction (*4*, *11*). A short, structurally defined α-helical motif in the CtIP-NTD is required for proper tetramerization, conveying an architecture essential for effective DSB repair by HR. CtIP mutation L27E blocks tetramerization and completely abrogates CtIP recruitment to DSB sites and HR (*8*). Overall, a DNA bridging mechanism was proposed, whereby CtIP helps to link DNA ends, thereby positioning its CTD close to the breaks to promote DNA end resection by the MRN complex (*12*, *13*). Accordingly, recombinant CtIP adopts a dumbbell-like structure, in which two globular DNA binding domains are held apart by a flexible “rod” corresponding to the coiled-coil domain of CtIP (*14*). Notably, *Schizosaccharomyces pombe* Ctp1 can form synaptic filamentous complexes on double-stranded DNA (dsDNA) and bridges dsDNA chains intra- or intermolecularly (*15*). Here, to establish proof of principle for therapeutic targeting of CtIP, we developed a cell-permeable constrained peptide inhibiting CtIP functions in genome maintenance by interfering with CtIP tetramer assembly.

## RESULTS

### Design of a hydrocarbon-stapled peptide mimicking and binding to the CtIP tetramerization motif

Tetramerization of CtIP is key to effective resection and homology-mediated repair of DSBs (*8*). Consequently, we sought to target CtIP’s “dimer-of-dimers” interface using an innovative peptide-based approach. Precisely, we aimed to design a cell-permeable constrained peptide mimicking the α-helical tetramerization motif. Hydrocarbon stapling constitutes the most successful and most widely used strategy to reinforce an α-helical conformation (*16*). Two olefin-bearing non-natural amino acids are incorporated in place of nonessential residues and cross-linked using ruthenium-catalyzed ring-closing olefin metathesis (*17*). It is noteworthy that hydrocarbon stapling of peptides increases both their proteolytic stability and membrane penetrance (*18*, *19*).

**Fig. 1:**
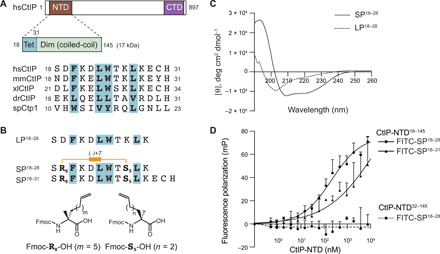
A CtIP-mimetic stapled peptide adopts an α-helical conformation and binds to the CtIP N terminus. (**A**) Top: Schematic drawing of full-length human CtIP (amino acids 1 to 897) harboring conserved NTDs and CTDs. The CtIP-NTD (amino acids 18 to 145) is divided into a tetramerization (Tet) motif (amino acids 18 to 31) and a coiled-coil region (amino acids 32 to 145) required for dimerization (Dim). Bottom: Multiple sequence alignments of the Tet motif in CtIP orthologs from *Homo sapiens* (hs), *Mus musculus* (mm), *Xenopus laevis* (xl), *Danio rerio* (dr), and *S. pombe* (sp). Conserved residues involved in α helix formation and required for tetramerization are highlighted in bold letters with blue boxes. (**B**) Design of hydrocarbon-stapled CtIP peptides. A staple was introduced by replacing D19 and K26 with olefin-bearing non-natural amino acids (R_8_ and S_5_) and subjecting the peptide to ruthenium-catalyzed ring-closing metathesis. In addition to a stapled peptide spanning the entire Tet motif (SP^18–31^), a shorter stapled version (SP^18–28^) and its linear counterpart (LP^18–28^) were synthesized. (**C**) CD spectra reveal enhanced α helicity for SP^18–28^ compared to LP^18–28^. (**D**) Fluorescence polarization binding curves of FITC-labeled SP^18–28^ and SP^18–31^ as a function of CtIP-NTD^18–145^ or CtIP-NTD^32–145^ concentration (mP, millipolarization units). The data are corrected for background by subtracting the free-labeled peptide background. Data are presented as means ± SEM (*n* = 3).

Multiple sequence alignment of the CtIP tetramerization motif (amino acids 18 to 31) among protein orthologs highlights an amphipathic two-turn helix with four highly conserved aromatic and hydrophobic residues (F20, L23, W24, and L27) packing against each other (Fig. 1A and fig. S1A) (*8*). To span these two helical turns and strengthen the bioactive α-helical conformation, we chose to apply an all-hydrocarbon i,i+7 stapling system (Fig. 1B) (*17*). Exclusion of peptide residues important for helical folding pointed toward a promising staple position between D19 and K26 (Fig. 1B). In addition to a stapled peptide spanning the entire tetramerization domain (SP^18–31^), we synthesized a shortened 11-mer stapled peptide lacking the C-terminal “ECH” sequence (SP^18–28^) and its linear counterpart (LP^18–28^) (Fig. 1B). We speculated that spontaneous oxidation of C30 could negatively affect peptide stability and conformation, while removing E29 results in a more positively charged peptide with potentially improved cellular uptake (*20*, *21*).

To evaluate the structural impact of hydrocarbon stapling, we performed circular dichroism (CD) measurements of SP^18–28^ and LP^18–28^ and could report a drastic increase in α-helical content upon stapling (Fig. 1C). Notably, the secondary structure in SP^18–28^ was profoundly heat stable, and no unfolding could be observed at temperatures of up to 90°C (fig. S1B). To assess the ability of our peptides to selectively bind the CtIP tetramerization motif, we purified recombinant CtIP-NTD^18–145^, folding into stable tetramers (*8*), and CtIP-NTD^32–145^, predominantly forming dimers (Fig. 1A and fig. S1C). Next, we measured binding affinities of fluorescein isothiocyanate (FITC)–labeled stapled peptides to CtIP-NTD^18–145^ using fluorescence polarization and observed a roughly 100-fold lower dissociation constant (*K*_d_) value for SP^18–28^ (208.7 nM) as compared to SP^18–31^ (Fig. 1D). Moreover, SP^18–28^ failed to bind CtIP-NTD^32–145^, indicating specific binding to the CtIP tetramerization motif (Fig. 1D).

### SP^18–28^ induces higher-order aggregation of CtIP

**Fig. 2:**
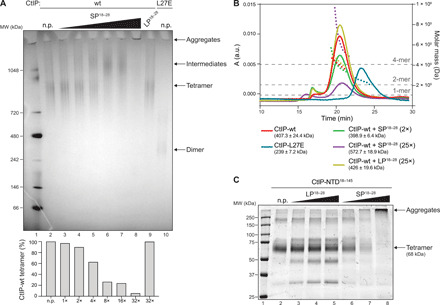
SP^18–28^ engages CtIP tetamers to form aggregates. (**A**) Recombinant full-length CtIP-wt was supplemented with increasing concentrations of SP^18–28^ (1-, 2-, 4-, 8-, 16-, and 32-fold molar excess) or LP^18–28^ (32-fold molar excess) and incubated for 30 min at RT before protein species were analyzed using blue native gel electrophoresis. In addition, blue native gel analysis of mock-treated (no peptide, n.p.) CtIP-wt and L27E are shown. Disappearance of CtIP tetramers was quantified by normalizing band intensities in each lane to n.p. (**B**) SEC-MALS analyses of CtIP-wt, CtIP-L27E, and of CtIP-wt following incubation with 2- and 25-fold molar excess of SP^18–28^ and LP^18–28^ are illustrated. Horizontal dashed lines depict the expected molecular weights (MW) for monomeric, dimeric, and tetrameric CtIP species. Dotted lines show the values for the fitted molecular masses. Calculated MW (kilodalton) of reactions are indicated in parentheses in the graph legend. When SP^18–28^ concentration was increased to 25 molar equivalents, extensive aggregation of CtIP-wt occurred as visible precipitation and is indicated by decreased UV absorption [A; arbitrary units (a.u.)]. (**C**) CtIP-NTD^18–145^ was either mock-treated (n.p.) or incubated with a 10-, 20-, or 50-fold molar excess of LP^18–28^ or SP^18–28^ for 30 min at RT. Thirty minutes after cross-linking, the samples were boiled and analyzed by SDS-PAGE and Coomassie staining.

We next subjected full-length recombinant CtIP proteins to native polyacrylamide gel electrophoresis (PAGE) and size exclusion chromatography coupled to multiple angle light scattering (SEC-MALS) analysis to determine CtIP oligomeric states upon incubation with the peptides. Under normal conditions, CtIP wild type (wt) and CtIP-L27E run as distinct single bands on native PAGE (Fig. 2A, lanes 2 and 10), and SEC-MALS shows that they adopt tetrameric and dimeric conformations, respectively (Fig. 2B), as previously established (*8*, *14*). Notably, CtIP tetramers run at anomalous positions relative to markers in both native PAGE and SEC (Fig. 2, A and B), most likely due to an estimated high content of intrinsic protein disorder that results in extended solution conformations in CtIP homologs (*12*). Incubation of CtIP-wt with LP^18–28^ caused no apparent structural changes (Fig. 2, A, lane 9, and B). Intriguingly, we observed higher–molecular weight species of CtIP-wt forming in the presence of increasing amounts of SP^18–28^ (Fig. 2A, lanes 4 to 7). When the SP^18–28^ concentration was increased to ≥25 molar equivalents, we noticed the formation of CtIP aggregates, manifesting as bands that do not enter the wells of native PAGE gels (Fig. 2A, lane 8) and visible precipitation of the CtIP sample. Accordingly, SEC-MALS–based molecular mass calculation of the CtIP molecules remaining soluble after filtration tended toward larger protein complexes in the presence of excess SP^18–28^ (Fig. 2B). Moreover, the smaller CtIP tetramer peak combined with an additional peak running at the void volume further corroborated elevated CtIP protein multimerization in the presence of SP^18–28^ (Fig. 2B). Similarly, SEC-MALS analyses of recombinant CtIP-NTD^18–145^ incubated with a 10-fold molar excess of SP^18–28^ showed a trend toward higher–molecular mass species (fig. S2A). Furthermore, SDS-PAGE analysis of CtIP-NTD^18–145^ products after cross-linking revealed that SP^18–28^ was able to induce the formation of high-order protein complexes in a concentration-dependent manner (Fig. 2C). SP^18–28^ did not hinder tetramerization-deficient CtIP-NTD^18–145, L27E^ and CtIP-NTD^32–145^ proteins from forming dimers but largely compromised the tetrameric configuration of CtIP-NTD^18–145^ (fig. S2B). Ctp1 in the fission yeast *S. pombe* has also been reported to feature an interlocking tetrameric helical dimer-of-dimers domain at its N terminus (*12*). In contrast to CtIP, however, we did not observe any major effects on Ctp1 structural integrity upon incubation with SP^18–28^ (fig. S3, A to C). Moreover, protein cross-linking experiments only revealed minor signs of Ctp1 aggregation tetramerization at the highest amount of SP^18–28^ (fig. S3D). Together, these findings established that a CtIP-mimetic stapled peptide specifically engages CtIP tetramers to agglutinate CtIP into higher-order multimers in vitro.

### CtIP-mimetic stapled peptides translocate to the nuclear compartment

**Fig. 3:**
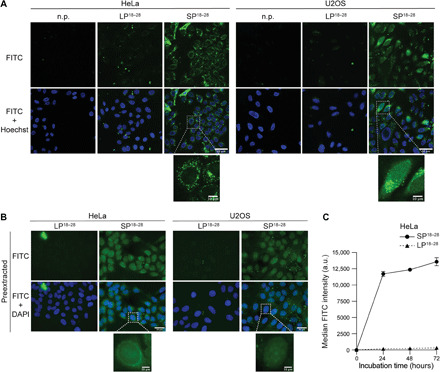
Hydrocarbon stapling mediates efficient intracellular delivery of SP^18–28^. (**A**) HeLa and U2OS cells were incubated with n.p. or 10 μM FITC-labeled peptides for 24 hours before staining with Hoechst 33342 and live-cell imaging using a confocal microscope. (**B**) HeLa and U2OS cells were incubated with 10 μM FITC-labeled peptides for 24 hours. Afterward, cells were thoroughly washed and preextracted before fixation and DAPI staining. Cells were imaged using a wide-field microscope. Scale bars, 50 and 10 μm (zoom). (**C**) HeLa cells were incubated with 10 μM FITC-labeled peptides for 24, 48, and 72 hours. Cells were harvested by trypsinization, and FITC signal was measured using flow cytometry. A total of 20,000 events were recorded. Graph depicts median FITC intensity (arbitrary units). Data are presented as means ± SD (*n* = 4).

Hydrocarbon stapling was previously shown to enhance cell penetration of peptides via endocytosis (*19*, *22*). Moreover, the staple introduced in our CtIP peptide is located at the amphipathic boundary, which has been previously found favorable for cellular uptake (*18*). Using live-cell confocal fluorescence microscopy, we observed efficient intracellular delivery of FITC-labeled SP^18–28^ into HeLa and U2OS cell lines, whereas LP^18–28^ did not cross the cell membrane barrier (Fig. 3A). The punctuate, predominantly cytoplasmic staining pattern suggested endocytosis as the primary uptake route with moderate nuclear staining (Fig. 3A). To verify whether SP^18–28^ was able to reach the nucleus, we preextracted HeLa and U2OS cells before image acquisition to remove the cytoplasmic compartment. We could detect homogenous nuclear SP^18–28^ staining in both cell lines (Fig. 3B). Next, we monitored FITC intensity in peptide-transduced HeLa cells over a time course of 3 days using flow cytometry to determine uptake efficiency and the intracellular residence time. SP^18–28^ was efficiently taken up during the first 24 hours with only a moderate increase in FITC intensity occurring during the following days (Fig. 3C). In contrast, we could not detect substantial amounts of intracellular LP^18–28^ (Fig. 3C). Moreover, we observed much lower FITC signals with SP^18–31^ compared to SP^18–28^ despite identical staple placement, indicating superior uptake or stability of the shorter peptide (fig. S4, A and B). Collectively, our findings suggested that stapling rendered the CtIP peptide membrane permeable with adequate SP^18–28^ levels reaching the nuclear compartment.

### SP^18–28^ impairs CtIP localization to DSBs and its ability to initiate DNA end resection

**Fig. 4:**
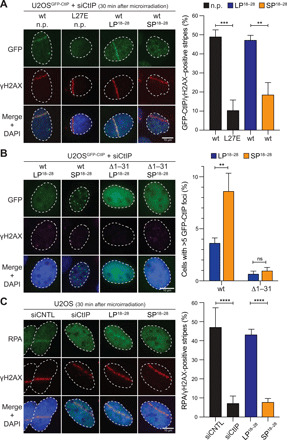
SP^18–28^-induced CtIP aggregates are defective in DNA end resection. (**A**) U2OS cells inducibly expressing siRNA-resistant GFP-CtIP-wt (U2OS^GFP-CtIP-wt^) or GFP-CtIP-L27E (U2OS^GFP-CtIP-L27E^) were transfected with siCtIP to deplete endogenous CtIP. The next day, cells were grown in the presence or absence of indicated peptides (10 μM), Dox, and BrdU for 24 hours before microirradiation. Cells were fixed 30 min after laser beam irradiation and immunostained for γH2AX before DNA staining with DAPI and fluorescence microscopy. Left: Representative images. Scale bar, 10 μm. Right: Graph depicts percentages of GFP-positive laser tracks relative to the total of γH2AX-positive stripes. Data, means ± SEM (*n* = 4). (**B**) U2OS^GFP-CtIP-wt^ and U2OS^GFP-CtIP-Δ1–31^ cells, inducibly expressing an N-terminally truncated CtIP variant lacking the tetramerization motif, were transfected with siCtIP to deplete endogenous CtIP. The next day, cells were grown in the presence of the indicated peptides (10 μM) and Dox for 24 hours. Cells were fixed, stained with DAPI, and imaged using a fluorescence microscope. Left: Representative microscopy images. Scale bar, 10 μm. Right: Graph shows percentage of cells with more than five GFP-CtIP foci. Data are presented as means ± SEM (*n* = 4). At least 250 cells were scored for each condition. (**C**) Parental U2OS were transfected with control (CNTL) or CtIP siRNA oligos for 48 hours. Alternatively, cells were treated with 10 μM of indicated peptides for 24 hours before microirradiation. Cells were grown for 24 hours in BrdU and, 30 min after irradiation, fixed and coimmunostained for RPA and γH2AX. DAPI was used to stain the DNA. Left: Representative microscopy images. Scale bar, 10 μm. Right: Graph illustrates the percentage of RPA-positive relative to γH2AX-positive laser tracks. Data, means ± SEM (*n* = 3). Statistical significance in (A) and (C) was calculated with Tukey’s multiple comparison test using one-way ANOVA. Statistical significance in (B) was calculated with Sidak’s multiple comparison test using two-way ANOVA. ****P ≤ 0.0001; ***P ≤ 0.001; **P ≤ 0.01; ns, nonsignificant.

CtIP tetramerization was shown to be required for efficient CtIP accumulation at laser-induced DNA damage sites and, consequently, DNA end resection (*8*). To investigate the phenotypic impact of SP^18–28^ on CtIP nuclear distribution and DSB resection, we generated stable U2OS clones inducibly expressing siCtIP-resistant green fluorescent protein (GFP)–tagged CtIP-wt or CtIP-L27E. As expected, CtIP-wt readily localized to microlaser-induced DSB tracks, whereas CtIP-L27E failed to do so (Fig. 4A). SP^18–28^ treatment severely impaired CtIP recruitment to γH2AX-decorated laser stripes, while LP^18–28^ had no significant effect (Fig. 4A). Following treatment with SP^18–28^, we frequently observed the formation of distinct nuclear GFP-containing focal structures, indicative of CtIP aggregation that is independent of DNA damage (Fig. 4B and fig. S5A). Notably, SP^18–28^ incubation failed to induce GFP-CtIP foci in cells expressing a CtIP mutant lacking the tetramerization motif (Fig. 4B and fig. S5A). In addition, endogenous CtIP was also prone to accumulate in focal structures upon SP^18–28^ treatment (fig. S5B). These findings suggested that cellular treatment with SP^18–28^ promotes the aggregation of CtIP tetramers, further corroborating our in vitro data. To examine whether these nonfunctional CtIP aggregates are more prone to being cleared by protein degradation, we performed Western blot analysis of time-course lysates from SP^18–28^-treated cells. However, we only observed a minor impact on steady-state levels of CtIP in a cycloheximide (CHX) chase experiment (fig. S5, C and D).

DNA end resection generates 3’ single-stranded DNA tails that are immediately coated by replication protein A (RPA) and subsequently replaced by RAD51 to form recombinogenic nucleoprotein filaments (*3*, *4*). To study the impact of SP^18–28^ on DNA end resection, we quantified RPA accumulation at γH2AX-decorated laser tracks. Intriguingly, upon treatment with SP^18–28^, U2OS cells displayed a pronounced decrease in RPA-positive stripes that was comparable to that observed in CtIP-depleted cells (Fig. 4C). As DNA end resection predominantly occurs during the S and G_2_ phases of the cell cycle (*23*), it was important to demonstrate that SP^18–28^ treatment neither interferes with bulk DNA replication nor causes any significant changes in cell cycle distribution and cell proliferation (fig. S5, E and F). From these analyses, we concluded that incubation of cells with SP^18–28^ compromised CtIP accumulation and resection at DSB sites, most likely due to the induction of dysfunctional higher-order CtIP complexes.

### SP^18–28^ interferes with homology-directed repair and replication fork protection

**Fig. 5:**
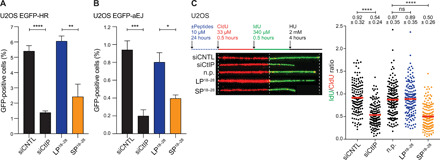
SP^18–28^ treatment compromises DSB repair and replication fork stability. (**A**) Forty-eight hours after siRNA transfection, U2OS EGFP-HR were transfected with *I-SceI* expression plasmid. Alternatively, cells were incubated with depicted peptides (10 μM) 4 hours after *I-SceI* transfection for 24 hours. Cells were harvested 48 hours after *I-SceI* transfection, and the percentage of GFP-positive cells was determined by flow cytometry. A minimum of 20,000 events were recorded. The graph depicts the percentages of GFP-positive cells as a readout for HR frequency. (**B**) U2OS EGFP-aEJ reporter cell line was transfected with *I-SceI* 6 hours after siRNA transfection. The next day, cells were treated with 10 μM LP^18–28^ or SP^18–28^ for 24 hours before harvesting the cells 72 hours after *I-SceI* transfection and subjecting them to flow cytometry. A minimum of 20,000 events were recorded. The percentage of GFP-positive cells was used as a readout for the frequency of a-EJ. Data in (A) and (B) are represented as means ± SEM (*n* = 4). Statistical significance was calculated with unpaired two-tailed *t* test (****P ≤ 0.0001; ***P ≤ 0.001; **P ≤ 0.01; *P ≤ 0.05). (**C**) U2OS cells were transfected with indicated siRNA oligos for 48 hours. Alternatively, cells were mock-treated (n.p.) or treated with 10 μM of the indicated peptides for 24 hours before pulse-labeling with CIdU/IdU for 30 min each and HU treatment (2 mM, 4 hours). Left: Schematic of labeling protocol to evaluate fork degradation is shown. In addition, representative images of CldU and IdU replication tracks are depicted. Right: Data are represented as scatterplot of IdU/CldU tract length ratios for individual replication forks. Numbers indicate the means ± SD. Statistical significance was calculated by Mann-Whitney test (****P ≤ 0.0001).

CtIP-mediated resection promotes error-free repair of DSBs by HR but, under certain conditions, also facilitates mutagenic repair by a-EJ implicated in tumorigenesis (*5*, *6*). To evaluate the effect of SP^18–28^ on HR and a-EJ, we took advantage of two established DSB reporter cell lines (*10*, *24*). We first analyzed HR efficiency of *I-SceI*–induced DSBs in U2OS cells following incubation with CtIP-mimetic peptides and observed a nearly threefold decrease in HR frequency in the presence of SP^18–28^ that was comparable to CtIP depletion, while LP^18–28^ had no measurable impact (Fig. 5A). Moreover, SP^18–28^ treatment caused a significant reduction in a-EJ frequency (Fig. 5B), reminiscent of what has been reported for tetramerization- and dimerization-deficient CtIP mutants (*8*, *10*).

Besides its well-established role in the processing of DSB ends together with MRN, CtIP was recently reported to protect stalled replication forks from DNA2-dependent nucleolytic degradation (*7*). To first assess whether CtIP tetramerization itself is required for fork protection in response to DNA replication stress, we first performed dual-labeling DNA fiber assays in U2OS^GFP-CtIP-wt^ and U2OS^GFP-CtIP-L27E^ cells treated with hydroxyurea (HU) to induce fork stalling (fig. S6). Consistent with a crucial function of CtIP tetramers in stabilizing stalled replication forks, we found that expression of CtIP-L27E failed to rescue nascent strand degradation in CtIP-depleted cells (fig. S6). We found that SP^18–28^ treatment promotes extensive fork degradation in U2OS cells, comparable to that observed upon CtIP depletion (Fig. 5C). SP^18–28^ treatment of U2OS^GFP-CtIP-L27E^ cells did not result in any additive increase in fork degradation (fig. S6), indicating that SP^18–28^ mediates its cellular activity largely through selective CtIP inhibition. Collectively, these findings further substantiate the use of SP^18–28^ as a potent and specific inhibitor of CtIP tetramerization and, consequently, of homology-directed DSB repair and fork protection.

### SP^18–28^ confers DNA damage hypersensitivity and selectively kills *BRCA1*-mutated cancer cells

**Fig. 6:**
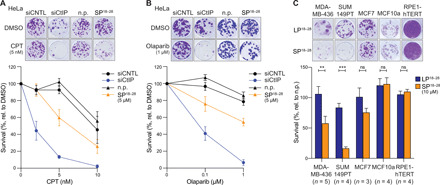
SP^18–28^ confers hypersensitivity to DNA-damaging agents and is toxic to *BRCA1*-mutated breast cancer cell lines. (**A**) HeLa cells were transfected with the indicated siRNAs or left untransfected for peptide treatment. Twenty-four hours later, cells were plated at low cell density into 24-well plates. Another 24 hours later, non-siRNA transfected cells were either mock-treated (n.p.) or treated with 5 μM SP^18–28^ together with increasing CPT concentrations and grown for 10 days. Top: Representative images of a colony formation assay are shown. Bottom: Clonogenic survival was determined by quantifying the colony intensity of CPT-treated relative to untreated cells. Data are presented as means ± SEM (*n* = 4). (**B**) The same cells as in (A) were treated with indicated olaparib concentrations with or without 5 μM SP^18–28^. Cells were grown for 10 days before fixation and staining with crystal violet. Top: Representative images of survival assay are depicted. Bottom: Clonogenic survival was determined by quantifying the colony intensity of olaparib-treated relative to untreated cells. Data are presented as means ± SEM (*n* = 5). (**C**) MDA-MB-436, SUM149PT, MCF7, MCF10a, and RPE1-hTERT were seeded at low cell density into 24-well plates and grown in the absence (n.p.) or presence of 10 μM LP^18–28^ or SP^18–28^ for 24 hours. After 10 days, cells were fixed, and colony intensities were measured. Top: Representative images of colony formation. Bottom: Graph illustrates survival by calculating the colony intensity of peptide-treated relative to mock-treated (n.p.) cells. Data are presented as means ± SEM. Statistical significance was calculated with Sidak’s multiple comparison test using two-way ANOVA (***P ≤ 0.001; **P ≤ 0.01). DMSO, dimethyl sulfoxide.

Given that unrepaired DSBs are highly cytotoxic, CtIP-depleted cells are hypersensitive to various DNA-damaging agents, most prominently to drugs inducing replication-associated DSBs, such as DNA interstrand–cross-linking drugs, DNA topoisomerase poisons [e.g., camptothecin (CPT)], and PARP1 inhibitors (e.g., olaparib) (*4*, *25*–*27*). Therefore, we next sought to examine the peptide’s potency to enhance cell death following treatment with anticancer drugs using clonogenic survival assays. Continuous exposure of HeLa cells to 5 μM SP^18–28^ considerably reduced colony formation when combined with increasing concentrations of either CPT or olaparib (Fig. 6, A and B). To more precisely determine the dose-dependent effects of acute versus chronic peptide treatment on DNA damage sensitivity, we performed clonogenic assays with HeLa cells grown in the absence or presence of olaparib (fig. S7A). While an acute (24-hour) treatment with 10 μM SP^18–28^ is well tolerated and resulted in significant olaparib hypersensitivity, a prolonged chronic treatment with the same dose caused extreme cytotoxicity on its own (fig. S7A). Consistent with data obtained from HeLa cells (Fig. 6A), chronic treatment with 5 μM SP^18–28^ significantly resensitized CtIP-wt– but not CtIP-L27E–expressing U2OS cells to CPT, indicating that peptide-mediated chemosensitization can be mainly attributed to the lack of functional CtIP tetramers (fig. S7B).

We have previously reported that combined deficiency of CtIP and BRCA1 synergistically compromises fork stability and provokes elevated levels of chromosomal instability in the absence of exogenous DNA damage (*7*). Furthermore, CtIP depletion drastically reduced the viability of a panel of *BRCA1* mutant breast cancer cell lines, implying a synthetic sick genetic interaction between these two factors (*7*). Thus, we speculated that SP^18–28^ treatment may be particularly toxic for BRCA1-deficient cells. SP^18–28^ incubation resulted in reduced clonogenic survival of MDA-MB-436 and SUM149PT *BRCA1* mutant breast cancer cell lines but not of BRCA1-proficient MCF7 breast cancer or MCF10A and RPE1 nontumorigenic cell lines (Fig. 6C). To exclude potential off-target effects of SP^18–28^ in inducing synthetic lethality in BRCA1-deficient cells, we used an isogenic MDA-MB-436 cell line pair (*28*) and observed decreased viability exclusively in control but not in *BRCA1* “add-back” cells following acute treatment with 10 μM SP^18–28^ (fig. S7C). Moreover, in response to CPT treatment, we noticed a considerable reduction in the phosphorylation levels of RPA2 and CHK1 specifically in SP^18–28^-treated MDA-MB-436 control cells (fig. S7D), indicative of impaired DSB resection (*4*). Overall, these findings support a scenario in which targeted CtIP inhibition is incompatible with viability when applied to BRCA1-deficient cells, likely due to severe combined deficiencies in DSB repair and fork protection.

## DISCUSSION

Cancer cells strongly rely on efficient mechanisms sensing and repairing different types of DNA damage to survive and proliferate. CtIP-mediated DNA end resection is required for DSB repair by HR to resist conventional DNA-damaging anticancer regimens (*2*). We reasoned that targeted inhibition of CtIP’s resection activity may provide a suitable approach for enhancing the efficacy of radio- and chemotherapy and may also be applied as monotherapy in certain genetic contexts based on the concept of synthetic lethality. Recent findings have established that CtIP and Ctp1 tetramerization, which is mediated via a structurally defined short α-helical motif in the N terminus, is crucial for its DNA bridging and repair function (*8*, *12*, *14*, *15*).

Here, we developed a peptide-based CtIP inhibitor targeting its tetramerization domain. Specifically, we reinforced an α-helical two-dimensional conformation by a constraining technique, referred to as hydrocarbon peptide stapling (*29*, *30*). We found that a peptide spanning CtIP residues 18 to 28 with an i,i+7 staple at locations 19 and 26 (SP^18–28^) was the best CtIP binder and interfered with CtIP tetramerization. The cell-permeable SP^18–28^ inhibited CtIP accumulation at DSB sites, causing defects in DNA end resection, HR, and fork protection and conferring hypersensitivity to DNA-damaging drugs. Most relevant from a clinical point of view, we observed SP^18–28^-mediated killing of *BRCA1*-mutated breast cancer cells, whereas no apparent toxicity was detected in nontumorigenic cell lines.

CtIP assembles as a dumbbell-like structure, whereby two C-terminal DNA binding domains are held apart by two coiled-coil domains (*14*). Intriguingly, the L27E point mutation not only impairs the dimer-of-dimers arrangement but also heavily reduces DNA binding (*14*). Consequently, targeting tetramerization may cause severe alterations in CtIP function. Biochemical evidence suggests that the tetramer seems to be the constitutive oligomeric state of CtIP (*13*). On the basis of our in vitro experiments, SP^18–28^ specifically binds the CtIP tetramerization domain, but instead of disrupting the tetramers into its dimeric or monomeric counterparts, the stapled peptide promotes extensive CtIP aggregation. Sae2, the yeast counterpart of CtIP, forms an inactive soluble multimeric complex during G_1_ phase and transitions to active oligomers upon extensive phosphorylation in S-G_2_ (*31*). Similarly, SP^18–28^ seems to trap CtIP in an inactive multimeric protein complex by an as-yet unknown mechanism. SP^18–28^ neither bound the N-terminal coiled-coil domain nor aggregated tetramerization-deficient mutants CtIP-NTD^18–145,L27E^ or CtIP-NTD^32–145^. Thus, we hypothesize that the CtIP-mimetic peptides can stably associate with the CtIP tetramerization motif and induce the formation of complex CtIP-peptide hetero-oligomers.

Several studies have shown that CtIP/Ctp1 promotes inter- and intramolecular DNA bridging (*12*, *14*, *15*). Notably, Andres and colleagues (*15*) reported that Ctp1-mediated DNA bridging relies on the formation of synaptic filaments involving, on average, 12 Ctp1 tetramers. These findings suggest that dynamic oligomerization states of Sae2/Ctp1 are critical for efficient DSB repair. However, no reports about the dynamic control of CtIP oligomerization in a biological context are available. It is conceivable that CtIP may be able to transit between different oligomeric conformations through distinct post-translational modifications or DNA binding as it was reported for Sae2/Ctp1 (*12*, *31*, *32*). Overall, our findings suggest that CtIP can adopt distinct conformational states, such as inactive higher-order multimers, to regulate DSB repair.

Hydrocarbon stapling frequently increases membrane penetrance, whereas native peptides commonly do not cross the cell membrane barrier (*33*). By hiding the hydrophilic peptide backbone and presenting the hydrophobic residues on one side, stapling may alleviate membrane crossing (*22*). Penetration mostly occurs through endocytosis, which conforms with our observation of intracellular punctuate staining patterns upon incubation with FITC-labeled SP^18–28^ (*22*, *34*). This uptake mode is significantly slower than direct transduction through the membrane, which is frequently observed with cell-penetrating peptides, and requires time to escape from endosomes (*34*). Although a major part seemed to be trapped in endosomes, a significant fraction of SP^18–28^ reached the nucleus and was sufficient to trigger a biological response. We speculate that the overall positive net charge of SP^18–28^ combined with an extended hydrophobic interface conferred the peptide a higher intracellular uptake rate than its longer counterpart SP^18–31^ (*18*).

Tetramerization-deficient CtIP mutants are defective in DNA end resection and homology-directed repair of DSBs most likely due to impaired dynamic CtIP redistribution at broken chromatin (*8*). Likewise, we observed a drastic decrease in CtIP redistribution to laser-induced DSBs and homology-mediated repair upon treatment with SP^18–28^. These findings were most likely related to the formation of higher-order CtIP oligomers with abrogated spatial redistribution to DSB sites. CtIP-L27E mutant is proficient in binding to MRN and undergoes phosphorylation despite its inability to accumulate at breaks (*8*, *35*).

Last, we report that inhibition of CtIP tetramerization interferes with its recently established role in the protection of reversed forks (*7*). The observed synergism between CtIP and BRCA1 in alleviating replication stress–induced chromosomal instability could be exploited by treating *BRCA1*-mutated cells with the CtIP inhibitor SP^18–28^ (*7*). We observed specific cell killing in a BRCA1-deficient background, whereas we did not perceive a negative impact on cell survival of *BRCA1* wt cell lines MCF7, MCF10A, and RPE1. Whether the lack of toxicity in MCF10A and RPE1 cells can solely be attributed to the presence of BRCA1 or whether it can also be partially explained by an overall decreased cellular uptake rate in normal versus cancer cells requires further investigation (*36*).

SP^18–28^ seems to be a potent inhibitor of functional CtIP oligomerization with a relatively low dissociation constant in the nanomolar range. Nevertheless, we had to provide peptide concentrations of 5 μM or higher to observe bioactivity. A major limitation of the stapled peptide is its predominant endocytic uptake resulting in high peptide amounts being trapped in endosomes. To transform this first-generation compound into a lead compound, diversification of staple type and position combined with fine-tuning core hydrophobicity and positive net charge is likely to increase cellular uptake and robust bioactivity (*37*, *38*). Prominent amino acid substitutions include the incorporation of arginines or arginine derivatives, namely homo-arginine and 4-guanidino-phenylalanine (*38*). Alternatively, complementary drug delivery strategies including the use of cell-penetrating peptides and nuclear localization signals could be used to improve cytoplasmic and nuclear uptake efficiencies (*36*, *38*).

Overall, SP^18–28^ constitutes a potent peptide therapeutic whose bioactive concentration range is comparable to successful peptidomimetics, including various p53/MDM2-targeting stapled peptides and a proapoptotic BH3-mimetic peptide (*19*, *21*). Intriguingly, clinical trials of ALRN-6924, a stapled peptide reactivating p53 expression, are underway and propose promising future applications of constrained peptides in the clinic (*39*). Notably, a recent report suggested that the extended drug interface of peptide therapeutics entails a higher resilience to point mutations in the target protein and impedes the acquisition of drug resistance (*40*).

We speculate that the stapled CtIP peptide inhibitor could have diverse areas of application in cancer therapy. In addition to conferring increased sensitivity to conventional DNA-damaging agents, CtIP-targeting therapeutics could be used in combination with PARPi in HR-proficient tumors. HR-mediated repair of DSBs predominantly operates during S phase of the cell cycle and is thus more significant for rapidly dividing cancer cells than for neighboring, healthy cells, providing a broad therapeutic window. Inhibition of mutagenic DSB repair by a-EJ may prolong the response of HR-deficient tumors to PARPi by preventing resistance acquisition (*41*). In addition to HR reactivation, restoration of fork protection seems to be a major mechanism conferring PARPi- and chemoresistance (*42*, *43*). Consequently, administration of SP^18–28^ would allow a two-pronged strategy for treating PARPi-resistant cancers by simultaneous disruption of HR and fork protection. Last, we provide evidence that SP^18–28^ can be applied in BRCA1-deficient tumors and is not toxic to noncancerous cell lines, which would allow selective tumor killing.

## MATERIALS AND METHODS

### Bacterial strains

CtIP-NTD^18–145^ and CtIP-NTD^32–145^ were expressed in electrocompetent BL21-CodonPlus-RIL *Escherichia coli*. XL1-blue competent *E. coli* cells were used for plasmid cloning.

### Cell culture

Human embryonic kidney 293 T, HeLa, U2OS, RPE1-hTERT [American Type Culture Collection (ATCC)], MCF7, U2OS EGFP-HR, and U2OS EGFP-aEJ cells were maintained in Dulbecco’s modified Eagle’s medium (DMEM) (Gibco, Thermo Fisher Scientific) supplemented with 10% fetal calf serum (FCS; Gibco, Thermo Fisher Scientific), penicillin (100 U/ml), and streptomycin (100 μg/ml) (1% P/S; Gibco, Thermo Fisher Scientific). U2OS^GFP-CtIP-wt^, U2OS^GFP-CtIP-L27E^, and U2OS^GFP-CtIP-Δ1–31^ cells were grown in DMEM containing 10% Tet system–approved FCS (Gibco, Thermo Fisher Scientific) and 1% P/S. GFP-CtIP expression was induced by providing doxycycline (Dox; 1 μg/ml; TaKaRa Clontech) for 24 hours. MDA-MB-436 (ATCC), MDA-MB-436 pLenti-IRES-GFP-BFP, and MDA-MB-436 pLenti-IRES-BFP-HABRCA1 (*28*) were cultured in RPMI 1640 medium (Gibco, Thermo Fisher Scientific) supplemented with 10% FCS, 2 mM l-glutamine, and 1% P/S. SUM149PT (BIOIVT) cells were maintained in HAM’s F12 medium (Gibco, Thermo Fisher Scientific) containing 10% FCS, 2 mM l-glutamine, and 1% P/S. MCF10A (ATCC) cells were cultured in DMEM/F12 (Gibco, Thermo Fisher Scientific) supplemented with 5% horse serum (Gibco, Thermo Fisher Scientific), human epidermal growth factor (20 ng/ml; Sigma-Aldrich), hydrocortisone (0.5 μg/ml; Sigma-Aldrich), insulin (10 μg/ml), and 1% P/S. All cell lines were grown at 37°C in an atmosphere containing 6% CO_2_.

### Peptides

Custom-designed peptides were purchased from Bachem AG (Bubendorf, Switzerland) and synthesized according to standard practice with N-terminal acetylation and C-terminal amidation. Alternatively, peptides were labeled with a fluorescein (FITC) tag at the N terminus. A comprehensive list with the exact peptide sequences can be found in table S1. Lyophilized LP^18–28^, SP^18–28^, and FITC-LP^18–28^ were dissolved in ddH_2_O at 1 mg/ml, whereas 1 mg/ml of FITC-SP^18–28^ and FITC-SP^18–31^ was solubilized in 50% dimethyl sulfoxide.

### siRNA and antibodies

Small interfering RNA (siRNA) sequences are listed in table S2. Transfections were performed with a final concentration of 10 nM using Lipofectamine RNAiMAX (Thermo Fisher Scientific) according to the manufacturer’s instructions. A detailed list of primary and secondary antibodies is provided in table S3.

### Recombinant protein expression and purification

CtIP-L27E mutation was introduced by site-directed mutagenesis of 3xFLAG-CtIP-wt expression vector (*8*) using primers listed in table S2. The CtIP-NTD ranging from amino acids 18 to 145 was polymerase chain reaction (PCR)–amplified from FLAG-CtIP-wt and L27E plasmids (primers, see table S2) and ligated into pET28 MBP-TEV vector (Addgene, #69929) upon restriction digest with Bam HI and Xho I (NEB). CtIP-NTD^32–145^ was generated by PCR of pET28-MBP TEV CtIP-NTD^18–145^ using 5′ phosphorylated primer “CtIP-NTD^32–145^ for” and “CtIP-NTD^32–145^ rev” (see table S2 for sequences) and subsequent plasmid religation.

CtIP-NTD fragments were purified as described previously (*11*). Briefly, constructs were expressed in *E. coli* BL21-CodonPlus-RIL for 20 hours at 18°C using 0.5 mM isopropyl-β-d-thiogalactopyranoside. Pellets were resuspended in lysis buffer [50 mM tris (pH 8.0) and 300 mM NaCl], snap-frozen, and thawed on ice. Subsequently, 1 mM phenylmethylsulfonyl fluoride, protease inhibitor cocktail (Roche), and lysozyme (0.1 mg/ml; Sigma-Aldrich) were added before stirring the bacterial lysates for 15 min at 4°C and sonicating them for 5 min. Insoluble material was removed by ultracentrifugation at 125,000*g* for 1 hour, and supernatant was loaded onto amylose affinity columns (5-ml MBPTrap HP, GE Healthcare). Fusion protein was eluted with 20 mM tris (pH 8.0), 2 mM β-mercaptoethanol (β-me), 300 mM NaCl, and 2 M methyl α-d-glucopyranoside (AMG; Sigma-Aldrich), and a buffer exchange into 20 mM tris (pH 8.0), 300 mM NaCl, and 5 mM β-me was performed using a HiPrep 26/10 Desalting column (GE Healthcare). N-terminal His_6_-MBP tag was removed by overnight cleavage at 20°C using MBP-tagged TEV protease (Gene and Cell Technologies) in a ratio of 1:5. TEV protease cleavage site products were captured by amylose affinity chromatography (5-ml MBPTrap HP, GE Healthcare). Further contaminants were removed by preparative SEC (HiLoad 16/600 Superdex 75, GE Healthcare) in 20 mM tris (pH 8.0), 150 mM NaCl, and 5 mM β-me. Full-length CtIP was purified according to (*14*). Full-length Ctp1^1–294^ and MBP-tagged Ctp1^1–60^ proteins were expressed and purified as described previously (*12*).

### Fluorescence polarization

CtIP-NTD^18–145^ or CtIP-NTD^32–145^ was serially diluted in black, flat-bottom 96-well plates (Greiner) in 20 mM tris (pH 8.0) and 150 mM NaCl. FITC-SP^18–28/18–31^ stock (1 mg/ml) was diluted at 1:10,000, and 50 μl of protein dilutions was mixed with 50 μl of FITC-labeled peptides before incubating for 10 min at room temperature (RT). Fluorescence polarization was recorded with an excitation wavelength of 470 nm, emission wavelength of 527 nm, 20-nm emission bandwidth, and 100 reads per well using a Tecan Safire 2 spectrometer. *K*_d_ values were calculated by nonlinear regression of dose-response curves.

### CD spectroscopy

CD spectroscopy data were collected on a Jasco J-710 spectropolarimeter. A total of 0.2 or 0.05 mg/ml of LP^18–28^ or SP^18–28^ in ddH_2_O was measured with a 1-mm path length cuvette. CD spectra were recorded at 0.5-nm intervals between 190 and 260 nm at 25°C. CD thermal denaturation data were recorded at 210 nm at 0.2°C intervals starting at 25° until 90°C.

### Blue native PAGE

Reactions were assembled in a volume of 10 μl with a 1.5 μM final concentration of CtIP and either 0, 1.5, 3, 6, 12, 24, or 48 μM LP/SP^18–28^ in buffer A [10 mM tris (pH 8.0), 25 mM NaCl, 5% glycerol, and 0.5 mM dithiothreitol (DTT)] and 1× Blue Native Loading buffer [50 mM bis-tris (pH 7.2), 6 N HCl, 50 mM NaCl, 10% (w/v) glycerol, and 0.001% Ponceau S]. After incubation for 30 min at RT, samples were loaded onto a NativePAGE Novex 3 to 12% bis-tris gel (Invitrogen) and run at 150 V for 75 min. Gels were fixed in 40% methanol (MeOH) and 10% acetic acid and destained with 8% acetic acid.

Ctp1 (1.5 μM) was incubated with SP^18–28^ for 30 min at RT in 10 mM tris (pH 8), 25 mM NaCl, 0.5 mM DTT, and 10% glycerol. Thereafter, 10 μl of each reaction was loaded onto NuPAGE 4 to 12% bis-tris gels (Invitrogen) that were then run at 150 V for 6 hours in 50 mM Mops and 50 mM tris base (pH 7.7) running buffer.

### Size exclusion chromatography coupled to multiple angle light scattering

SEC-MALS was used to determine the absolute molecular mass of full-length CtIP in the presence and absence of LP^18–28^ and SP^18–28^. Reactions were assembled in a total volume of 100 μl with a 10 μM final concentration of CtIP and either 0, 20, or 250 μM peptides in buffer A [12 mM tris-HCl (pH 8.0), 150 mM NaCl, 6% glycerol, and 0.6 mM DTT]. These were incubated on ice for 1 hour and then at RT for 30 min before being loaded at 0.5 ml/min onto a Superose 6 10/300 SEC column (GE Healthcare) in 20 mM tris (pH 8.0), 200 mM NaCl, and 0.5 mM Tris (2-Carboxyethyl)-Phosphin (TCEP) using an Agilent high-performance liquid chromatography (HPLC). The eluate from the column was coupled to a DAWN HELEOS II MALS detector (Wyatt Technology) and an Optilab T-rEX differential refractometer (Wyatt Technology). ASTRA 6 software (Wyatt Technology) was used to collect and analyze light scattering and differential refractive index data according to the manufacturer’s instructions. Molecular masses and estimated errors were calculated across individual eluted peaks.

An Agilent 1100 HPLC was used to inject 100 μl of CtIP NTD^18–145^ (2 mg/ml), CtIP NTD^18–145^ L27E (2 mg/ml), and CtIP NTD^32–145^ (8 mg/ml) individually onto a WTC-030S5 size exclusion column (Wyatt Technology) with a 1 ml/min flow rate in running buffer [20 mM tris (pH 8), 150 mM NaCl, and 2 mM β-me running buffer]. CtIP NTD^18–145^ (20 μM) was incubated with 200 μM SP^18–28^ for 30 min on ice and then centrifuged for 10 min at high speed to remove excess aggregates before injection of 100 μl. The eluate from the column was coupled to a μDAWN MALS detector (Wyatt Technology) and an Optilab RI detector (Wyatt Technology). ASTRA 7 software (Wyatt Technology) was used to collect and analyze light scattering and differential refractive index data according to the manufacturer’s instructions. Molecular masses and estimated errors were calculated across individual eluted peaks.

SEC of Ctp1 proteins was performed on a Superdex S200 increase 10/300 GL column (Cytiva) in 20 mM tris (pH 8), 150 mM NaCl, and 2 mM β-me. A total of 32 μM Ctp1 ± 5-fold excess of SP^18–28^ or 10 μM MBP-Ctp1^1–60^ ± 10-fold excess of SP^18–28^ was incubated for 1 hour at 4°C before precipitation being spun down and 100 μl being injected onto the column.

### Protein cross-linking

Recombinant proteins (1 or 2 μg) were mixed with peptides in 1× phosphate-buffered saline (PBS) and incubated at RT for 30 min with gentle shaking. Chemical cross-linking was carried out with 100 μM disuccinimidyl suberate (Sigma-Aldrich) at RT for 30 min with gentle shaking. Cross-linking reactions were quenched by the addition of 50 mM tris (pH 7.5) for 5 min at RT before boiling in 1× SDS sample buffer [5 mM tris (pH 6.8), 10% glycerol, 1.6% SDS, 100 mM DTT, and 0.02% bromophenol blue] for 5 min. Protein samples were separated by SDS-PAGE, and the gels were stained with InstantBlue (Expedeon).

### Generation of U2OS GFP-CtIP Flp-In T-REx cells

Mammalian expression vector pcDNA5/FRT/TO-GFP-CtIP L27E was generated by site-directed mutagenesis of pcDNA5/FRT/TO-GFP CtIP-wt using primers listed in table S2. Expression vector pcDNA5/FRT/TO-GFP-CtIP Δ1–31 was generated by PCR linearization of pcDNA5/FRT/TO-GFP-CtIP-wt using 5′ phosphorylated primers “CtIP del1-31 for” and reverse primer “CtIP del rev” (table S2). Subsequently, plasmids were religated using T4 DNA ligase (NEB). U2OS cell lines stably expressing siRNA-resistant GFP-CtIP-wt, L27E, or Δ1–31 were generated using the Flp-In T-REx system as described previously (*44*). Precisely, U2OS Flp-In T-REx cells were transfected with pcDNA5/FRT/TO-GFP-CtIP constructs and Flp recombinase expression plasmid pOG44 (1:9 ratio) using Lipofectamine 3000 transfection reagent (Invitrogen) according to the manufacturer’s instructions. Forty-eight hours after transfection, cells were plated at varying densities, and 6 hours later, cell selection was performed by supplementing medium with hygromycin B (250 μg/ml; InvivoGen) and blasticidin S (15 μg/ml; InvivoGen) for 14 days. Single-cell clones were picked and analyzed for GFP expression by immunoblotting, flow cytometry, and immunofluorescence microscopy. To induce GFP-CtIP expression, cells were grown for 24 hours in Dox (1 μg/ml).

### Confocal and immunofluorescence microscopy

Cells were seeded into eight-well chamber imaging slides (Ibidi) and grown overnight. Upon treatment with 10 μM FITC-labeled peptides for 24 hours, cells were imaged in Live Cell Imaging Medium (Gibco, Thermo Fisher Scientific) containing Hoechst 33342 (0.5 μg/ml; Thermo Fisher Scientific) using CLSM SP5 Mid UV-VIS Leica with 63× objective at 37°C and ambient CO_2_ concentrations. Nuclear peptide uptake was evaluated by washing cells twice with cold PBS before preextraction for 5 min on ice [25 mM Hepes (pH 7.4), 50 mM NaCl, 1 mM EDTA, 3 mM MgCl_2_, 300 mM sucrose, and 0.5% Triton X-100], fixation with 4% formaldehyde (w/v) in PBS for 15 min at RT, and imaging with Leica DM6, 63× objective.

Laser microirradiation was carried out as described previously (*25*). Briefly, cells were grown in 10 μM 5-bromo-2′-deoxyuridine (BrdU) for 24 hours before irradiation. Laser microirradiation was performed using a MMI CELLCUT system containing an ultraviolet (UV) A laser of 355 nm. Energy output was set to 50%, and each cell was exposed to laser beam for <300 ms. Cells were released for 30 min before fixation in 4% formaldehyde (w/v) in PBS for 15 min and permeabilization with 0.5% Triton X-100 (w/v) in PBS for 5 min at RT. After blocking with 3% FCS (w/v) in PBS for 1 hour, cells were stained with primary antibodies (table S3) for 2 hours. Staining with secondary antibodies (table S3) was performed for 1 hour. Coverslips were mounted with Vectashield supplemented with DAPI (4′,6-diamidino-2-phenylindole; Vector Laboratories), and images were acquired on a Leica DM6, 63× objective. For CtIP foci analyses, cells were fixed in 4% formaldehyde (w/v) in PBS for 15 min before permeabilization with 0.5% Triton X-100 (w/v) in PBS for 5 min at RT. A blocking step with 3% FCS (w/v) in PBS for 1 hour was followed by primary (2-hour) and secondary (1-hour) staining at RT.

### Immunoblotting

Cells were lysed in Laemmli buffer [4% SDS, 20% glycerol, and 120 mM tris (pH 6.8)], resolved by SDS-PAGE, and transferred to nitrocellulose membranes. Immunoblotting was performed with indicated primary antibodies (table S3) overnight at 4°C and horseradish peroxidase–conjugated secondary antibodies (table S3) for 1 hour at RT. Proteins were visualized using the Advansta Western Bright enhanced chemiluminesence reagent (Advansta) and Fusion Solo S imaging system. For the CHX chase assay, U2OS cells were either mock-treated or incubated with the indicated peptides for 24 hours and afterward treated with CHX (50 μg/ml; Sigma-Aldrich) for 0, 2, or 6 hours before cell lysis.

### Flow cytometry analysis

HeLa cells were seeded into six-well plates and incubated with peptides in a volume of 2 ml for varying time points. Cells were harvested by trypsinization, washed, and resuspended in PBS before subjecting them to flow cytometry analysis. FITC intensity was measured with an Attune Nxt flow cytometer equipped with a 488-nm laser and 530/30 band-pass filter. Analysis of 5-Ethynyl-2′-deoxyuridine (EdU) incorporation was carried out using the Click-iT EdU technology (Thermo Fisher Scientific) as described in the manufacturer’s instructions. A minimum of 20,000 events were recorded.

### Cell proliferation assay

A total of 125 and 250 U2OS cells were seeded in triplicates in 96-well plates in a volume of 90 μl. Twenty-four hours after seeding, 10 μl of medium only or medium with the indicated peptides was added to the wells reaching a final peptide concentration of 5 μM. Cell proliferation was monitored using a CellTiter-Blue–based (Promega) approach at specific time points (0, 2, 5, and 7 days after peptide treatment). In brief, 20-μl CellTiter-Blue reagent (Promega) was added to the wells, incubated for 4 hours, and fluorescence intensity was measured at 560/590 nm using a SpectraMax M5 microplate reader (Molecular Devices).

### HR and a-EJ reporter assay

HR reporter assay was performed as described previously (*45*, *46*). Briefly, U2OS EGFP-HR were seeded into a 12-well plate and the day after transfected with *pCBA I-SceI* expression plasmid using jetPRIME transfection reagent (Polyplus). Four hours later, medium was exchanged, and cells were incubated with peptides for 24 hours in 1-ml total volume. Twenty-four hours later, medium was replaced, and cells were harvested 48 hours after *I-SceI* transfection. A-EJ reporter assay was performed according to (*10*) with some minor modifications. Specifically, U2OS EGFP-aEJ were seeded into six-well plates and siRNA transfected. Six hours later, cells were transfected with *I-SceI* expression plasmid (pCBA) using Fugene 6 (Promega). Medium was exchanged 24 hours after and replaced with 2-ml fresh medium and the peptides (10 μM). Twenty-four hours later, medium was again replaced with fresh medium without peptides, and 72 hours after *I-SceI* transfection, cells were harvested. GFP expression (readout for HR and a-EJ frequency) was measured by flow cytometry using the Attune Nxt flow cytometer equipped with a 488-nm laser and 530/30 band-pass filter. A minimum of 20,000 events were recorded.

### DNA fiber analysis

DNA fiber analysis was carried out according to (*7*). U2OS cells were plated into six-well plates and incubated with 2 ml of medium/peptide mix. Twenty-four hours later, U2OS cells were labeled with 33 μM CldU (Sigma-Aldrich) for 30 min, 340 μM IdU (5′-iododeoxyuridine; Sigma-Aldrich) for 30 min, and followed by treatment with 2 mM HU (Sigma-Aldrich) for 4 hours. Cells were lysed [200 mM tris (pH 7.4), 50 mM EDTA, and 0.5% SDS], DNA was stretched onto glass slides, and fibers were fixed in MeOH:acetic acid (3:1). Rehydration with PBS was followed by denaturation with 2.5 M HCl for 1 hour and a PBS wash. DNA fibers were blocked in 2% bovine serum albumin and 0.1% Tween 20 (w/v) in PBS for 40 min. CldU/IdU tracks were immunostained using anti-BrdU primary and corresponding secondary antibodies for 2.5 hours each (see table S3). Coverslips were mounted using ProLong Gold Antifade Mountant (Life Technologies), and images were acquired on a Leica DM6 microscope, 63× objective. DNA fiber lengths were analyzed using Fiji software.

### Clonogenic survival assay

Two hundred cells per well were seeded into poly-l-lysine (Sigma-Aldrich)–coated 24-well plates and the next day treated with respective drugs (CPT, Sigma-Aldrich; olaparib, Selleck Chemicals) and peptides (total volume: 300 μl per well). For details, see figure legends. After 10 days of growth, cells were fixed with crystal violet solution [0.5% crystal violet and 20% ethanol (w/v)]. Plates were scanned, and survival was analyzed with the ImageJ plugin Colony Area using the parameter colony intensity as readout (*47*).

### Quantification and statistical analysis

Statistical analyses were performed using GraphPad Prism. Statistical tests are reported in the figure legends. If not indicated otherwise, each experiment was repeated at least three times. If the data conformed to a normal distribution, then an unpaired two-tailed *t* test was used. One-way analysis of variance (ANOVA) and Tukey’s multiple comparison test were used when comparing multiple groups with each other. Two-way ANOVA and Sidak’s multiple comparison test were applied to compare multiple groups of two factors. Fiber experiments were performed twice (*n* = 2), and representative experiments are depicted. Samples were subjected to a Mann-Whitney analysis. *P* values of <0.05 were considered statistically significant. ns, not significant; *P ≤ 0.05; **P ≤ 0.01; ***P ≤ 0.001; ****P ≤ 0.0001. Fluorescence polarization was fitted with nonlinear regression using the model *Y* = *B*_max_ * *X^h^* / (*K*_D_*^h^* + *X^h^*), where binding at equilibrium by *B*_max_ is the maximum specific binding, *K*_D_ is the ligand concentration needed to achieve a half-maximum binding at equilibrium, and *h* is the Hill slope.

## SUPPLEMENTARY MATERIALS

Supplementary material for this article is available at http://advances.sciencemag.org/cgi/content/full/7/8/eabc6381/DC1

## Appendix

View/request a protocol for this paper from *Bio-protocol*.
